# Ultrafast non-radiative dynamics of atomically thin MoSe_2_

**DOI:** 10.1038/s41467-017-01844-2

**Published:** 2017-11-23

**Authors:** Ming-Fu Lin, Vidya Kochat, Aravind Krishnamoorthy, Lindsay Bassman Oftelie, Clemens Weninger, Qiang Zheng, Xiang Zhang, Amey Apte, Chandra Sekhar Tiwary, Xiaozhe Shen, Renkai Li, Rajiv Kalia, Pulickel Ajayan, Aiichiro Nakano, Priya Vashishta, Fuyuki Shimojo, Xijie Wang, David M. Fritz, Uwe Bergmann

**Affiliations:** 1 Linac Coherent Light Source, SLAC National Accelerator Laboratory, Menlo Park, CA 94025 USA; 2 Stanford PULSE Institute, SLAC National Accelerator Laboratory, Menlo Park, CA 94025 USA; 3 Department of Materials Science and NanoEngineering, Rice University, Houston, TX 77005 USA; 4 Collaboratory for Advanced Computing and Simulations, Department of Physics & Astronomy, Department of Computer Science, Department of Chemical Engineering & Materials Science, Department of Biological Sciences, University of Southern California, Los Angeles, CA 90089-0242 USA; 5 SLAC National Accelerator Laboratory, Menlo Park, CA 94025 USA; 6 Department of Physics, Kumamoto University, Kumamoto, 860-8555 Japan

**Keywords:** Molecular dynamics, Two-dimensional materials

## Abstract

Photo-induced non-radiative energy dissipation is a potential pathway to induce structural-phase transitions in two-dimensional materials. For advancing this field, a quantitative understanding of real-time atomic motion and lattice temperature is required. However, this understanding has been incomplete due to a lack of suitable experimental techniques. Here, we use ultrafast electron diffraction to directly probe the subpicosecond conversion of photoenergy to lattice vibrations in a model bilayered semiconductor, molybdenum diselenide. We find that when creating a high charge carrier density, the energy is efficiently transferred to the lattice within one picosecond. First-principles nonadiabatic quantum molecular dynamics simulations reproduce the observed ultrafast increase in lattice temperature and the corresponding conversion of photoenergy to lattice vibrations. Nonadiabatic quantum simulations further suggest that a softening of vibrational modes in the excited state is involved in efficient and rapid energy transfer between the electronic system and the lattice.

## Abstract (web summary)

Knowledge of the energy transfer pathways in transition metal dichalcogenides is essential to design efficient optoelectronic devices. Here, the authors use megaelectronvolt ultrafast electron diffraction to unveil the sub-picosecond lattice dynamics in MoSe_2_ following photoexcitation of charge carriers

## Introduction

Energy loss pathways are important considerations for the design of optoelectronic devices based on nanomaterials like transition metal dichalcogenides (TMDCs)^1,2^, which are promising candidates for ultrafast photodetection^3^, valleytronics^4^, and field effect transistors^5,6^. Much of the recent attention has been focused on the radiative relaxation and defect-mediated non-radiative bimolecular recombination of excitons, which have been shown to drastically limit photoluminescence quantum yield even at the monolayer limit (~0.4%)^7–12^. In addition, considerable effort has been expended in the strain engineering^13,14^ and in identifying strategies for chemical passivation of defects to improve light emission efficiency and device performance^15,16^. The nonradiative channel is the dominant energy loss pathway in defect-free monolayers at high charge carrier densities but is less well explored^1,6,9,15^. Recent work from Ruppert et al.^17^ has shown that an optically excited monolayer WS_2_ relaxes nonradiatively to lattice vibration, causing the absorption edge to redshift due to a small temperature jump of ~40 K. This nonradiative process is promising for triggering light-driven atomic motion required for structural-phase transition, but so far, it is not well understood due to a lack of experimental methods to quantify atomic motion and lattice temperature directly.

In this study, we utilize mega-electronvolt ultrafast electron diffraction (MeV-UED)^18^ as a probe to measure mean-square atomic displacements and the corresponding local temperature in bilayer MoSe_2_ samples photoexcited to a high carrier density (~10^14^ cm^−2^), which lies in the electron–hole plasma regime. At this high charge carrier concentration, the laser fluence of the optical pump is high enough to deposit the energy required for initiating a possible structural-phase transition (temperature jump of ~1000-K and ~0.3-Å difference of lattice constant between 2-H and 1-T phases). Time-resolved electron diffraction allows us to directly explore laser-induced atomic motion and structural disorder with ~200-fs temporal resolution, and thus to directly quantify the efficiency of nonradiative energy channels in these two-dimensional materials. We monitor the dependence of the resulting lattice disorder on the photoinjected carrier concentration by varying the pump fluence at two different wavelengths. The observed subpicosecond increase in structural disorder and lattice temperature is consistent with ultrafast conversion of electronic energy to atomic motion via softened phonon modes observed in our first-principles density functional theory (DFT) and nonadiabatic quantum molecular dynamics (NAQMD) simulations. Furthermore, the observed ultrafast temperature rise is linearly proportional to the absorption of the pump energy calibrated to a saturable absorber model, indicating a high efficiency of the nonradiative decay channel.

## Results

### Time-resolved electron diffraction

**Fig. 1:**
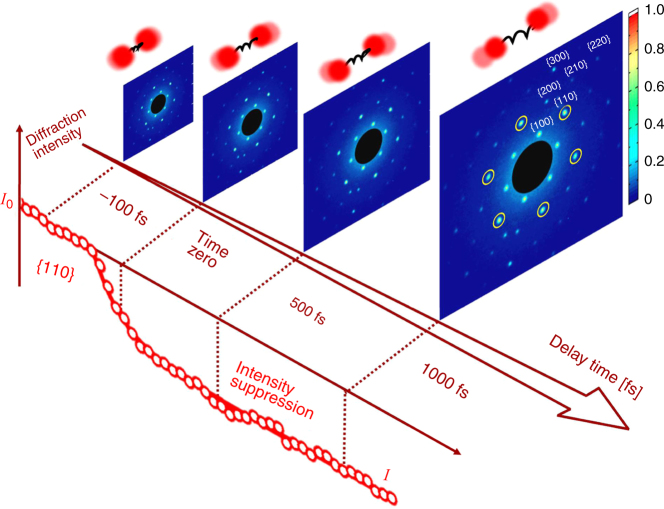
Time-resolved mega-eV electron diffraction. Snapshots of electron diffraction of MoSe_2_ bilayer for Debye–Waller factor (DWF) measurements. Several families of diffraction planes are labeled in the last diffraction image for clarity (i.e., {100}, {110}, {200}, {210}, {300}, and {220}). A time-resolved kinetic plot that illustrates the suppression of diffraction intensity at {110} family is shown, where *I*_0_ and *I* denote the Bragg peak diffraction intensities at negative and positive delay times from the fit of experimental results. Each image is accumulated over ~7000 pulses from multiple scans with effective charge of ~20 fC per pulse at the sample target. The mean-square displacements, $\left\langle {\Delta u^{\mathrm{2}}} \right\rangle _{\Delta T}$, are obtained by plotting ${\mathrm{ln}}\left( {\frac{{I_0}}{I}} \right)$ vs. *Q* ^2^, where *Q* is 2*π* over interplanar spacing *d*

A schematic of the experimental procedure and a representative electron diffraction pattern and intensity profile is displayed in Fig. 1. Each diffraction pattern is generated by the accumulation of over ~7000 pulses with effective charge of ~20 fC per pulse at the sample target. Several families of diffraction planes in the reciprocal lattice are observed. All the pump-probe traces at a given reciprocal lattice vector **Q** (i.e., 2*π*/*d*) are averaged over six equivalent planes. Diffraction intensity for a lattice plane is calculated from the full diffraction pattern by the integration of photodetector counts on a small region around the corresponding reciprocal lattice vector. Negative delay times are shown when the electron probe beam precedes the optical pump beam on the sample target. A red kinetic plot in Fig. 1 displays a representative temporal profile of the measured diffraction intensity, which shows a rapid exponential decay of the initial diffraction intensity value *I*_0_ to a new lower value of *I* at a positive delay time due to the increased incoherent atomic motion induced by the optical pumping. The ratio of these two intensities allows us to estimate the atomic disorder and the resulting temperature jump produced by the optical excitation, which will be further discussed later in this paper.

### Theoretical approach

Our UED experiments are further supported by NAQMD simulations on MoSe_2_ bilayer crystals, which follow the trajectories of all atoms while computing interatomic forces quantum mechanically from first principles^19^, including the dynamics of electrons and nuclei^20^. Electronic excitations are described within the framework of linear-response time-dependent DFT, which is a proven method to investigate ultrafast nonradiative relaxation in other electronically excited material systems^21^. In this study, nonadiabatic transitions between electronic excited states assisted by molecular motions are treated with a surface-hopping approach.

In order to elucidate atomic dynamics observed in our QMD simulations, we also calculated phonon dispersion curves for the ground-state and electronically excited MoSe_2_ bilayers using DFT and the Δ-SCF method. Specifically, for excited electron–hole concentration of 1.2 × 10^14^ cm^−2^ (2.4 × 10^14^ cm^−2^) used in this study, we promote one (two) electrons from the valence band to the conduction band. The comparison of experimental results from Si_3_N_4_-supported MoSe_2_ flakes to simulation results from suspended MoSe_2_ bilayers is justified for two reasons. First, MoSe_2_ bilayer forms a type-I heterojunction with the insulating Si_3_N_4_ substrate (~5-eV bandgap), which precludes a charge transfer process. Second, Raman microscopy and photoluminescence measurements from the Si_3_N_4_-supported MoSe_2_ agree with previous measurements on different substrates^22^, indicating a lack of substrate effect on MoSe_2_ vibration modes.

### Debye–Waller response of bilayer MoSe_2_

**Fig. 2:**
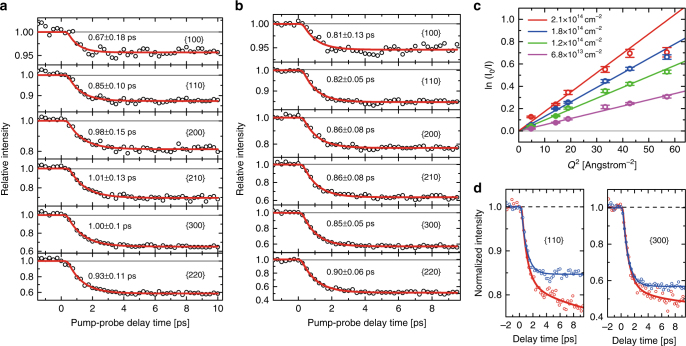
Pump-probe kinetics and Debye–Waller responses at 400-nm photoexcitation. Pump-probe kinetic plots for six diffraction planes at a carrier density of (**a**) 1.2 ± 0.1 × 10^14^ cm^−2^ and (**b**) 1.8 ± 0.1 × 10^14^ cm^−2^ at 400 nm excitation for the MoSe_2_ bilayer, and (**c**) Debye–Waller responses at four carrier densities. A large deviation between linear fit and experimental data occurs at the highest carrier density (red). At this density, the first five data points are used for the linear fit. The corresponding temperatures calculated from the slopes are presented in the context and Supplementary Information. Error bars represent 68% confidence interval and are calculated from the propagation of standard error of the mean from the fit of averaged multiple pump-probe scans. **d** Kinetic plots for {110} and {300} planes at carrier density at 1.8 ± 0.1 × 10^14^ cm^−2^ (blue) and 2.1 ± 0.1 × 10^14^ cm^−2^ (red), respectively, for comparison. A complex Bragg peak intensity decay occurs at the highest carrier density (red), which may suggest an energy redistribution between different phonons from a strong carrier-modified potential energy surface

Figure 2 summarizes our experimental results for bilayer MoSe_2_ samples photoexcited at 400 nm. The temporal evolution of the measured diffraction intensities for six diffraction families at carrier densities of 1.2 ± 0.1 × 10^14^ cm^−2^ and 1.8 ± 0.1 × 10^14^ cm^−2^ is shown in Fig. 2(a, b), respectively. Note that all the pump-probe kinetics are normalized to one at the negative delay time (*I*_0_), so that the fractional drop of intensity can be quickly identified for different diffraction families. Each pump-probe trace is then fit to an exponential function convoluted with an ~200-fs full-width at half-maximum instrument response function (time resolution) estimated for the MeV-UED system. Thus, the obtained *I* and *I*_0_ from the fit are the relative electron diffraction intensities at far positive and negative delay times, respectively, as shown in the red curves in Fig. 2a, b. The kinetic plot at a lower carrier density of 0.68 ± 0.1 × 10^14^ cm^−2^ is presented in Supplementary Fig. 2a in Supplementary Note 3. The standard deviation error bars are approximately 0.04 by using multiple scans with a total of ~7000 pulses per delay time point. The primary observation in the intensity profiles is the ultrafast subpicosecond decay of diffraction intensities for all families of planes considered in our study. This is approximately a factor two faster than the time constant reported for the monolayer MoS_2_ at a similar carrier density (*τ* ~ 1.7 ps)^23^. Furthermore, the invariant steady-state diffraction intensity up to ten picoseconds indicates that the lattice is thermalized for delay times longer than one picosecond after optical excitation. The experimental time constants do not show any obvious changes as a function of the carrier density produced at 400 nm and 800 nm, implying a minor role of high-order carrier kinetics in these carrier density regimes.

Below, we consider three possible mechanisms to explain the ultrafast appearance of lattice disorder. First, the lattice energy could result from the high-order kinetics such as nonradiative bimolecular and Auger recombination through annihilation of photoexcited excitons. The obtained carrier density for this experiment is ~10^14^ cm^−2^ such that an electron–hole plasma is produced from screened exciton dissociation (i.e., the Mott density is ~10^14^ cm^−2^)^24–27^. It has been reported that the bimolecular exciton–exciton annihilation or Auger process mediated by defects/traps occurs efficiently in MoSe_2_ and MoS_2_^7,8,28^. However, this happens at exciton densities below ~10^13^ cm^−2^ allowing intact excitons to annihilate each other in the time scale of a few picoseconds. At a higher charge carrier density (~10^14^ cm^−2^), excitons dissociate into free carriers due to the increased screening of Coulomb interaction in the bound charges^24,27^. Therefore, the exciton–exciton annihilation may not be able to account for this fast appearance of lattice disorder. Our power dependence measurement at 400 nm does not show clear changes of time constants as a function of carrier density in the electron–hole plasma regime. This suggests that the high-order carrier kinetics such as Auger processes does not play an important role in our experiments.

The second possible explanation for the rapid decay of diffraction intensity may be due to the induction of nonthermal melting. Figure 2c shows the logarithmic ratios of measured diffraction intensities vs. the diffraction families in the square-reciprocal space at four carrier densities (i.e., ln(*I*_0_/*I*) vs. *Q* ^2^). The effective mean-square displacements associated with different carrier concentrations can be estimated from the slope^29,30^, $\Delta \left\langle {u^2} \right\rangle _{\Delta T}$, by using the Debye–Waller equation as shown in Eqs. (1) and (2).

1 $$I = I_0e^{ - \Delta \left\langle {u^2} \right\rangle _{\Delta T} \times Q^{\mathrm{2}}}$$

2 $${\mathrm{ln}}\left( {I_0/I} \right) = \Delta \left\langle {u^{\mathrm{2}}} \right\rangle _{\Delta T} \times {\it{Q}}^{\mathrm{2}}$$

The calculated mean-square displacements for the four different carrier concentrations are 5.6(±0.2) × 10^−3^ Å^2^, 9.8(±0.3) × 10^−3^ Å^2^, 13.0(±0.4) × 10^−3^ Å^2^, and 17.4(±1.2) × 10^−3^ Å^2^, respectively, corresponding to a root-mean-square atomic displacement, $\sqrt {\Delta \left\langle {u^2} \right\rangle _{\Delta T}}$, of 0.075 ± 0.001 Å, 0.099 ± 0.002 Å, 0.114 ± 0.002 Å, and 0.132 ± 0.005 Å. These photoinduced atomic displacements range from 2% to 5% of the in-plane lattice constant of 3.3 Å^31^, which is below the Lindemann criterion for a solid-to-liquid phase transition (i.e., ~10%–20%)^32^. In addition, these displacements are much less than what would be expected for highly inertial atomic motion initiated by an intense optical excitation (highly nonthermal melting)^33–35^. We estimate a (3*k*_B_ *T/m*)^1/2^ = 2.9 Å per picosecond mean atomic velocity for MoSe_2_ at room temperature, where *k*_B_, *m*, and *T* are the Boltzmann constant, averaged mass, and room temperature, respectively. At this velocity, the mean atomic displacements would be 1.45 Å at a 500-fs time delay, corresponding to ~44% of in-plane lattice constant. This is ~10 times higher than our observations at the highest pump fluence. Thus, our data are not consistent with a nonthermal melting and strong atomic inertial processes from potential deformation by the strong laser field.

Another possibility is the existence of strong electron–phonon coupling, which has been postulated for molybdenum chalcogenides with dynamics occuring on a subpicosecond time scale^36^. Figure 2d presents a comparison of kinetic plots for {110} and {300} planes at two carrier densities (i.e., 1.8 × 10^14^ cm^−2^ (blue) and 2.1 × 10^14^ cm^−2^ (red)). For the lower carrier density, a fast subpicosecond single-exponential decay of the diffraction intensity is observed. For the higher carrier density, we see an additional slower decay with a lifetime of ~10 ps in addition to the fast decay. Such a complex decay of diffraction intensity was previously reported for another layered material, graphite, where it was attributed to the coexistence of electron–phonon and phonon–phonon scattering, as well as to electron–phonon coupling to two different vibration modes^29,30^. In our experiments, the observed crossover from single exponential to biexponential intensity decay with increasing excited charge carrier concentration (and correspondingly different potential energy surfaces) is indicative of electron–phonon coupling to one or more decay channels at low and high charge carrier densities, respectively. The other diffraction planes also reveal two contributions from the fast and the slow decay channels, and the results are presented in Supplementary Fig. 2b.

### NAQMD simulation results

**Fig. 3:**
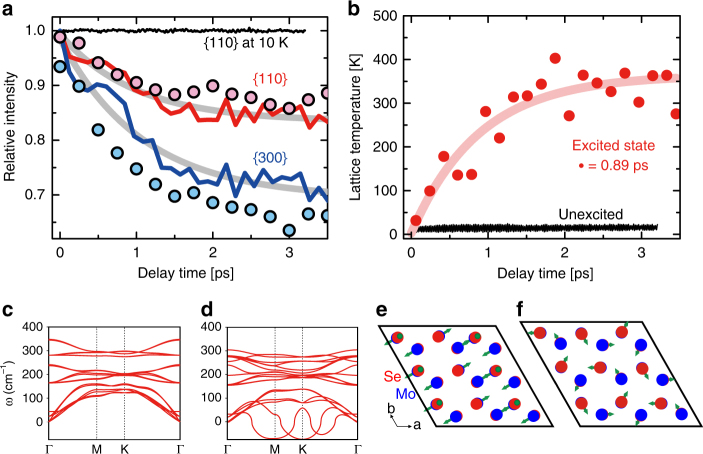
NAQMD simulations of bilayer MoSe_2_. **a** Debye–Waller factor for the MoSe_2_ bilayer as a function of delay time for the {110} and {300} planes calculated from NAQMD simulations at carrier density of 0 cm^−2^ (black) and 1.2 × 10^14^ cm^−2^ (red and blue lines). Photoexcitation results in a marked increase in structural disorder and a reduction in the Bragg peak intensity. Exponential fits to the simulations yield a time constant of ~1 ps, as shown in the gray curves. The experimental results shown in circles are the bilayer MoSe_2_ at a carrier density of 1.2 × 10^14^ cm^−2^. Note that there are no normalization factors between the experimental and simulation results. **b** Instantaneous temperature in the NAQMD simulation cell due to photoexcitation at time t = 0 shows a transient temperature increase from 10 K to 300 K over 1.5 ps. **c**, **d** display phonon dispersion curves for the MoSe_2_ bilayer at the photoexcited carrier density of **c** 0 cm^−2^, (**d**) 1.2 × 10^14^ cm^−2^, respectively. **e**, **f** Eigenvectors for the soft acoustic modes at the M-point and K-point, respectively

To interpret this ultrafast subpicosecond change of Bragg peak intensity as a function of delay time, we performed NAQMD simulations of MoSe_2_ bilayer in the electronically ground and excited states, respectively. Figure 3a shows the Debye–Waller factors (DWF) for the {110} (red) and {300} (blue) families of planes calculated from an ensemble of four photoexcited NAQMD atomic trajectories and compared to the Debye–Waller factors for the unexcited MoSe_2_ bilayer at 10 K. Single-exponential decay fits to the {110} and {300} planes show an ultrafast increase in structural disorder with time constants of ~1 ps (gray), and magnitude of DWF decay (Δ = 0.15 and 0.3) that are qualitatively consistent with experimental measurements (circles). This subpicosecond dynamics is further supported by Fig. 3b, which shows a plot of the instantaneous temperature of atoms in the simulation cell as a function of time. The calculated temperature of the simulation cell increases from the initial value of 10 K to a steady-state value of ~350 K in a lifetime scale of ~0.89 ps. Snapshots of time-resolved atomic velocity distributions at two delay times are presented in Supplementary Fig. 3 and Supplementary Note 4 to rationalize a fast equilibration of lattice temperature in our experiments.

To further understand the underlying ultrafast atomistic mechanisms, we calculated phonon dispersion curves as a function of electronic excitation with the method described previously for the MoSe_2_ bilayer. This allows us to study the effect of excited charge carrier population on the potential energy surface of the crystal. Figure 3c, d shows the phonon dispersion of the MoSe_2_ bilayer in the ground and excited states, respectively. Electronic excitation causes two primary differences to the potential energy surface of the crystal. First, the frequencies of atomic vibrations decrease uniformly throughout the Brillouin zone with increasing excited charge carrier concentration, reflecting a weakening of interatomic bonds leading to greater atomic displacements. Second, with increased charge carrier concentration, softening of zone-edge phonons is observed at the M (0.5,0,0) and K (0.33,0.33,0) points in the Brillouin zone. Eigenvectors corresponding to these soft modes are shown in Fig. 3e, f, respectively. The flattening of the potential energy surface corresponding to the soft vibration mode at the M-point enables in-plane displacement of Mo ions to form Mo–Mo dimer chains and is particularly important for displacements corresponding to the 2H → 1-T’ phase transition of MoTe_2_^37,38^. Such soft modes represent an instability in the 2-H crystal structure, but do not necessarily imply a spontaneous structural transformation from the 2H to the 1-T’ crystal structure, which would require, in addition, barrierless motion of Mo and Se atoms over 0.3 Å from the 2H to the 1-T’ lattice positions. Further, the largest observed RMS displacement in our pump-probe experiments is 0.13 Å prior to the onset of irreversible sample damage, which is significantly smaller than 0.3 Å required for a structural-phase transformation. However, the existence of such softened vibration modes induced by carrier population is an indication of strong electron–phonon coupling which could enable efficient relaxation of electronic energy to the lattice^39,40^. Phonon softening at M and K points in electron-doped MoS_2_ crystals was accompanied by large electron–phonon coupling constants at these *q*-points^41^. Similarly, in materials like amorphous bismuth and gallium, the large range of motion enabled by the soft vibrations gives rise to anomalously high electron–phonon coupling constants^42^.

### Debye–Waller responses at 800-nm photoexcitation

**Fig. 4:**
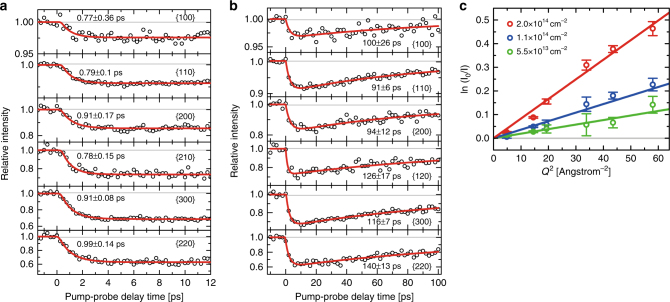
Pump-probe kinetics and Debye–Waller responses at 800 nm photoexcitation. **a** Time-resolved kinetic plots of MoSe_2_ bilayer for six diffraction families, as depicted in the parenthesis at a carrier density of 2.0 ± 0.2 × 10^14^ cm^−2^ excited at 800 nm. The resultant subpicosecond time constants are consistent with measurements at 400 nm. **b** At the same carrier density and wavelength for the bilayer with the pump-probe scan up to 100 ps. A clear recovery of Bragg peak intensity is shown (~100 ps). This is due to the heat propagation from the bilayer MoSe_2_ to the substrate. **c** Power-dependent Debye–Waller responses of a bilayer for three effective carrier densities at 800 nm photoexcitation. Error bars represent 68% confidence interval and are obtained from the propagation of standard error of the mean from the fit of averaged multiple pump-probe scans. The mean-square displacements obtained from the linear least-square fit of data are shown in Supplementary Table 3. Note that at the highest carrier density, the Debye–Waller response is still linear without a large deviation, as seen in Fig. 2c

This strong electron–phonon coupling enables a very efficient relaxation of excited carrier energy to the lattice disorder, and this suggests that in the electron–hole plasma regime, the transient lattice temperature rise is simply proportional to the absorbed pump energy. In order to understand the impact of excited charge carrier energy on lattice dynamics, we compare the measured lattice disorder produced by the optical pumping at 400 nm to results by 800-nm photoexcitation. In particular, at 800-nm excitation, the photon energy is half of a 400-nm photon. This allows us to produce ~50% of lattice disorder and temperature jump in comparison to 400-nm photoexcitation at a similar carrier density. Indeed, from our experiments at 800 nm, we observed a strong laser-driven Bragg peak intensity decay implying that the lattice disorder results from the electron–hole plasma-driven and phonon-softening nonradiative process. The kinetics at two different pump-probe delay ranges at 800-nm photoexcitation are shown in Fig. 4a, b, respectively. We chose similar carrier densities at 800 nm compared to 400 -nm experiments. In Fig. 4a, the resulting fast time constants are on subpicosecond time scales without any apparent *Q* ^2^ and carrier density dependence, consistent with the observation at 400-nm photoexcitation. This implies that the nonthermal melting and high-order carrier kinetics do not play an important role at 800-nm photoexcitation in this carrier density regime. Note that in our experiments, the irreversible sample damage from the accumulation of quasi-stationary heating does not exist because the heat propagation to the PMMA and silicon nitride substrate occurs on a time scale of 100 ps, as shown in Fig. 4b ^23^, which is eight orders of magnitude shorter than the time between electron pulses (i.e., 1/180 s). This ensures that the sample target equilibrates to room temperature before the next pump-probe cycle. This is very different from a recent experiment using Raman microscopy equipped with a CW and high-repetition-rate lasers at a diffraction-limit focus (~1 µm^2^)^43^. In that experiment, the average pump power was much higher than in the experiment presented here (i.e., 1000 W per cm^2^ vs. 1 W per cm^2^), but the peak power on the other hand is much lower. This indicates that the Raman experiments are mostly susceptible to the damage from the continuous heating, while for higher peak power, the damage results from single-shot ablation. Figure 4c shows the Debye–Waller responses as a function of carrier density at 800-nm photoexcitation, which allows us to measure mean-square displacements of a bilayer, as described previously at 400-nm photoexcitation.

### Temperature jump from UED measurements and correlation plots

We have also performed power dependence measurements and NAQMD simulations of monolayer MoSe_2_ samples, as displayed in Supplementary Figs. 4 and 5 in Supplementary Notes 5 and 6, respectively. We observed qualitatively similar time constants, Debye–Waller responses, and mean-square displacements as seen in the bilayer case. A comparison of experimental results between the monolayer and bilayer is shown in Supplementary Table 3 and Supplementary Note 2. In order to calculate the temperature jump of MoSe_2_ from the measured mean-square displacement, we apply an equation that employs Debye model with assumptions of harmonic approximation and isotropic vibration distribution with a cutoff frequency for the estimation of density of state^44^. This allows us to condense the integration of various phonon modes down to a simple analytical equation, as shown in Eqs. (3)–(5)

3 $$\Delta \left\langle {u^{\mathrm{2}}} \right\rangle _{\Delta T} = \left\langle {u^{\mathrm{2}}} \right\rangle _T - \left\langle {u^{\mathrm{2}}} \right\rangle _{{\mathrm{298K}}}$$

4 $$\left\langle {u^{\mathrm{2}}} \right\rangle _T = \frac{{3\hbar ^{\mathrm{2}}}}{{mk_{\mathrm{B}}\theta _{\mathrm{D}}}}\left[ {\left( {\frac{T}{{\theta _{\mathrm{D}}}}} \right)^{\mathrm{2}}P_{(\theta _{\mathrm{D}}/T)} + \frac{{\mathrm{1}}}{{\mathrm{4}}}} \right]$$

5 $$P_{(\theta _{\mathrm{D}}/T)} = \mathop {\int }\nolimits_0^{\frac{{\theta _{\mathrm{D}}}}{T}} \frac{x}{{e^x - 1}}{\mathrm{d}}x \cong 1.6449(1 - e^{ - 0.64486\frac{{\theta _{\mathrm{D}}}}{T}}),$$

where *ħ*, *m*, *k*_B_, and *θ*_D_ are the reduced Planck constant, averaged molecular mass, Boltzmann constant, and Debye temperature of the crystal, respectively. Here, we use 84.63 g per mole and 193 K for the averaged molar mass and Debye temperature, respectively^45^. Equation (4), with function $P_{\left( {\theta _{\mathrm{D}}/T} \right)}$, can be solved analytically from the known Debye temperature, as shown in Eq. (5)^46^. The efficiency of optical energy conversion into the thermal motion of a lattice is estimated by a comparison of temperature jump measured from the UED experiments to temperature increase calculated using absorption of the pump beam, the specific heat of the MoSe_2_ (S = 0.278 J per g per K)^47^, sample density, and volume. Detailed estimation of the temperature jumps from the absorption is presented in Supplementary Note 1. The calculated temperature jumps in a bilayer using Eqs. (3)–(5) are 116 ± 4 K, 203 ± 7 K, 269 ± 9 K, and 359 ± 25 K, respectively, corresponding to the temperatures of 414 ± 4 K, 501 ± 7 K, 567 ± 9 K, and 657 ± 25 K at four carrier densities at 400-nm optical excitation. At similar carrier densities, optical excitation by the 400-nm pump results in a larger transient lattice temperature jump than an 800-nm pump. The measured and estimated temperatures from UED and absorption, respectively, are shown in the following section. This correlation between UED results and effective absorption allows us to directly quantify the efficiency of the nonradiative channel.

**Fig. 5:**
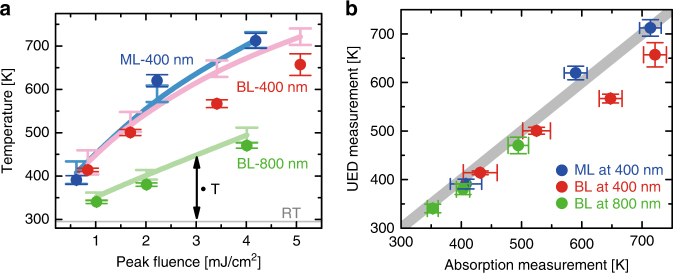
Temperature correlation plots for atomically thin MoSe_2_. **a** Temperatures of atomic thin MoSe_2_ from UED results (solid circles) and from effective absorption calculated from a saturable absorber model (solid lines). ΔT denotes the temperature jump above room temperature (RT). ML and BL symbolize the monolayer and bilayer, respectively. Error bars in UED results denote 68% confidence interval and are obtained from propagation of standard error of the mean from the fit of averaged multiple pump-probe scans. **b** Correlation plot between UED results and estimates from effective absorption. The gray solid line denotes a full linear correlation, implying a unity of nonradiative quantum yield to lattice disorder. Horizontal error bars in the absorption measurements are calculated by taking into account the uncertainties of powermeter measurements and saturation intensities in ref. ^48^

Figure 5a displays UED-measured temperatures (i.e., solid circles) as a function of pump fluence. A saturable absorber model of MoSe_2_ is used to calculate the effective absorption^48^, and the corresponding temperatures (i.e., solid lines) are plotted with the UED results (circles). We note that the reported saturation intensities (*I*_s_) from Wang et al.^48^ are used. The predicted temperature jumps from the saturated absorption model agree qualitatively with our UED measurements. We observe a slightly faster temperature rise for the monolayer (ML) than for the bilayer (BL). This can be rationalized as an absorption difference per unit layer between the monolayer and bilayer. In addition, photoexciting the bilayer at 800 nm reveals roughly half the temperature jump in comparison to the bilayer at 400 nm. This is a result of half the energy per 800-nm photon compared to that absorbed per 400-nm photon.

Figure 5b displays a correlation plot between temperature changes from UED results and from estimated absorption. The experimental results from UED correlate qualitatively with the estimated temperature jump from a saturable absorber model. From this correlation, we conclude that at the high carrier density, the average quantum yield of the nonradiative process approaches unity (0.87 ± 0.11). Most of the absorbed photon energy converts to lattice vibrations within a picosecond through strong electron–phonon interaction, and this lattice disorder recovers quickly by transporting energy to the substrates on the ~100-ps time scale. In our study, the maximum displacement prior to damage is only ~5% of the in-plane lattice constant, which is still lower than the reported theoretical value of ~11.5% for a phase transition to metallic MoSe_2_^49^.

## Discussion

In summary, we have utilized MeV UED to directly probe lattice dynamics of mono- and bilayer MoSe_2_. At electron–hole densities in the plasma regime, the nonradiative channel of laser-driven MoSe_2_ reaches near-unit efficiency with a maximum atomic displacement of ~5% of the lattice constant. The lattice motions are proportional to the injected carrier density estimated from the reported saturable absorber model. The subpicosecond suppression of Bragg peak intensity and the corresponding disorder of lattice planes are supported by first-principles NAQMD simulations, indicating that the electronic excitation-induced phonon softening is a precursor to efficient dissipation of electronic energy to lattice vibrations. This strong lattice response to optical excitations has important implications for light-induced semiconductor-to-metal structural-phase transition pathways in transition metal dichalcogenides.

## Methods

### Sample preparation

Bilayer single-crystal MoSe_2_ samples were prepared by chemical vapor deposition (CVD) on Si/SiO_2_ substrates at 750 °C and characterized by micro-Raman spectroscopy and photoluminescence measurements (see Supplementary Fig. 6 and Supplementary Note 7 for details), which are consistent with other reported values on different substrates^22^. The MoSe_2_ samples were transferred onto a ten-nanometer-thick silicon nitride membrane employing a PMMA-assisted transfer technique.

### Ultrafast electron diffraction

Diffraction experiments were performed at MeV-UED facility at SLAC National Accelerator Laboratory^18^. The pulsed electron beam was operated at a repetition rate of 180 Hz and an energy of 3.2 MeV. The beam with an ~40 µm rms spot size impinged on the sample in a normal incidence transmission geometry perpendicular to the (001) plane, therefore probing only in-plane lattice displacements^29^. The pulse duration and intensity of the electron beam were ~200 fs and ~20 fC, respectively. The MoSe_2_ bilayers were photoexcited with short optical laser pulses at 400 nm (3.1 eV) producing hot carriers, and at 800 nm (1.55 eV) producing band edge carriers. Charge carrier densities shown in all figures were estimated by taking into account the absorption cross section, reflectivity of MoSe_2_, and interfaces between the sample, PMMA, and silicon nitride membrane on a stack geometry of materials, as shown in Supplementary Fig. 1. The final absorption was then calibrated to a saturable absorber model, as shown in Supplementary Eq. 1 in Supplementary Note 1 ^48^. The obtained absorption, reflectivity, carrier density, and temperature jump are displayed in Supplementary Tables 1 and 2.

### Nonadiabatic quantum molecular dynamics

NAQMD simulations are performed on a MoSe_2_ bilayer supercell containing 54 atoms, corresponding to 3 × 3 unit cells of the 2-H ground-state crystal structure. Electronic states were calculated using the projector-augmented-wave (PAW) method, and projector functions were generated for 4*d*, 5*s*, and 5*p* states of Mo, and 3*d*, 4*s*, and 4*p* states of Se. The generalized gradient approximation (GGA) was used for the exchange-correlation energy with nonlinear core corrections. The GGA functionals used in this study do not include Hubbard on-site interaction correction. We have performed additional NAQMD simulations to confirm that the inclusion of the on-site interaction term (*U* = 2 eV) does not alter the time constant for lattice thermalization more than 0.01 ps nor the magnitude of atomic vibrations after excitation significantly. Van der Waals interactions were incorporated based on the DFT-D method. Plane-wave cutoff energies were 25 and 250 Ry for electronic pseudo-wave functions and pseudo-charge density, respectively. The energy functional was minimized iteratively using a preconditioned conjugate-gradient method. More details on the implementation of our QMD program can be found in ref. ^20^.

### Density functional theory

DFT calculations were performed on a 3 × 3 supercell of the 2H-MoSe_2_ bilayer crystal structure containing 54 atoms. Valence electron wave functions are constructed using a plane-wave basis set containing components up to a kinetic energy of 450 eV, and the reciprocal space is sampled using a 7 × 7 × 3 Monkhorst–Pack ***k***-point mesh with a Gaussian smearing of orbital occupancies of 0.1 eV. The Hessian matrix was generated using density functional perturbation theory, and dispersion relations for normal vibration modes of the MoSe_2_ unit cell were plotted using the open-source phonopy package^50^. For phonon dispersion calculations, excited states are described within the Δ-SCF DFT framework. In this scheme, a non-Aufbau constraint is imposed on orbital occupancies to promote electrons from the occupied Kohn–Sham energy levels to unoccupied higher-energy levels followed by relaxation of nuclear positions to minima in the potential energy surface defined by the charge density corresponding to the fixed occupancy of these energy levels. The Δ-SCF method is known to provide an inexpensive approximation to the potential energy surface of the excited-state system with accuracy comparable to time-dependent DFT methods^51,52^.

### Data availability

The data that support the findings of this study are available from the corresponding author upon request.

## Electronic supplementary material

Supplementary Information
