# Author Correction: Ultrafast non-radiative dynamics of atomically thin MoSe_2_

**DOI:** 10.1038/s41467-023-40538-w

**Published:** 2023-08-15

**Authors:** Ming-Fu Lin, Vidya Kochat, Aravind Krishnamoorthy, Lindsay Bassman Oftelie, Clemens Weninger, Qiang Zheng, Xiang Zhang, Amey Apte, Chandra Sekhar Tiwary, Xiaozhe Shen, Renkai Li, Rajiv Kalia, Pulickel Ajayan, Aiichiro Nakano, Priya Vashishta, Fuyuki Shimojo, Xijie Wang, David M. Fritz, Uwe Bergmann

**Affiliations:** 1 Linac Coherent Light Source, SLAC National Accelerator Laboratory, Menlo Park, CA 94025 USA; 2 Stanford PULSE Institute, SLAC National Accelerator Laboratory, Menlo Park, CA 94025 USA; 3 Department of Materials Science and NanoEngineering, Rice University, Houston, TX 77005 USA; 4 Collaboratory for Advanced Computing and Simulations, Department of Physics & Astronomy, Department of Computer Science, Department of Chemical Engineering & Materials Science, Department of Biological Sciences, University of Southern California, Los Angeles, CA 90089-0242 USA; 5 SLAC National Accelerator Laboratory, Menlo Park, CA 94025 USA; 6 Department of Physics, Kumamoto University, Kumamoto, 860-8555 Japan

Correction to: *Nature Communications* 10.1038/s41467-017-01844-2, published online 23 November 2017

In this article the author name Lindsay Bassman Oftelie was incorrectly written as Lindsay Bassman. The original article has been corrected.

