# Supplementary material for: Ultrafast non-radiative dynamics of atomically thin MoSe2
Source: Nat Commun. 2017 Nov 23;8:1745. doi: 10.1038/s41467-017-01844-2 (PMC5701075; doi:10.1038/s41467-017-01844-2)
Supplement: Supplementary file 1 — Supplementary Information [file 41467_2017_1844_MOESM1_ESM.pdf]

Supplementary Note 1. Temperature Jumps from Effective Absorption of Pump Fluences

Temperature jumps in atomic thin $MoSe_2$ are estimated using a model at normal incidence configuration as described in Supplementary Figure 1.[1] The complex refractive indices for $MoSe_2$, PMMA and Si_3N_4 membrane are 2.91-2.20i, 1.51 and 2.07 at 400 nm, and 4.06-0.82i, 1.49 and 2.01 at 800 nm, respectively.[2–4] PMMA residue and silicon nitride substrate are transparent at 400 nm and 800 nm excitation, thus their temperature jumps from absorption are ignored. At 400 nm excitation of bilayer (monolayer), we obtained 13 % (11.5 %) of reflection, 77.3 % (83.3 %) of transmission and 9.7 % (5.2 %) of absorption using the equations described in **4**(119) and **4**(120) in Supplementary Reference 1. This equation estimates the reflection, transmission and absorption from a stack of materials with different refractive indices.

Supplementary Table 1 presents the calculated reflection, transmission and absorption of the monolayer and bilayer $MoSe_2$ at 400 nm and 800 nm, respectively, in the assumption of one-photon absorption. The thicknesses of monolayer and bilayer used in the calculation of absorption are 0.65 nm and 1.3 nm, respectively.[5] Note that the residue of PMMA on top of $MoSe_2$ varies from 5 nm to 20 nm after acetone removal. However, its thickness effect on the absorption of $MoSe_2$ is minute (±0.2%) due to transparency of PMMA at 400 nm and 800 nm.

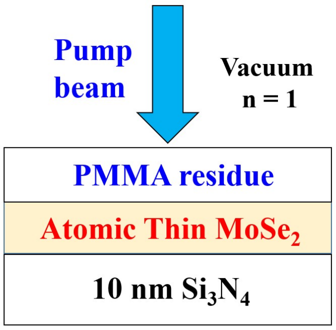

Supplementary Figure 1 | Normal incidence configuration of laser to the sample.

Normal incidence configuration of the pump laser to the atomic thin $MoSe_2$ sandwiched between PMMA residue (~20 nm) and silicon nitride membrane (10 nm). We are able to calculate the absorption, transmission and reflection from the known complex refractive indices of sample, substrates and surroundings (i.e. vacuum).[1] These results are shown in Supplementary Table 1.

Supplementary Table 1. Complex refractive indices and estimated absorption, reflection and transmission at 400 nm and 800 nm for monolayer and bilayer $MoSe_2$.

	Complex refractive index	Thickness (nm)	Absorption (%)	Reflection (%)	Transmission (%)
Monolayer 400 nm	2.91 - 2.20 i	0.65	5.2	11.5	83.3
Bilayer 400 nm	2.91 - 2.20 i	1.30	9.7	13.0	77.3
Bilayer 800 nm	4.06 - 0.82 i	1.30	2.8	13.1	84.1

Furthermore, $MoSe_2$ behaves as a saturable absorber in current pump fluence regions.[6] The effective absorption is further calibrated using Supplementary Equation (1), which takes in to account the saturation peak intensity (I_s) from femtosecond pulses. Here, we use 43±2 GW/cm^2 and 590±225 GW/cm^2 for 400 nm and 800 nm, respectively, as reported in the reference.[6] Supplementary Equation (1) writes

$$\alpha = \frac{\alpha_0}{1+\frac{I}{I_s}} \qquad (1)$$

, where α and α_0 represent the absorption cross sections with and without saturation effect of the pump beam. I and I_s denote the irradiated and saturated peak intensities, respectively. The final effective absorbed photon number leading to the temperature jumps, ΔT, are calculated using Supplementary Equation 2 from the known specific heat of $MoSe_2$, excitation area of the pump beam and sample thickness.

$$\Delta H = m \times S \times \Delta T \qquad (2)$$

, where ΔH, m and S are the absorbed pump energy, effective mass on the pump area and specific heat, respectively. In addition, carrier densities are calculated in the assumption of one-photon absorption. All estimated values such as temperature jumps, effective mass and carrier densities at different pump fluences and wavelengths are presented in Supplementary Table 2. Density and specific heat of $MoSe_2$ are 6.96 g per cm^3 and 0.278 J per g per K (at 300 K) for the estimation of T jump.[7] The estimated pump area is ~0.01 cm^2 with 20% variation from different experimental conditions. We note that the final temperature in the film is equal to the sum of room temperature and ΔT. The peak fluence is calculated using equation $F(mJ\ per\ cm^2) = 2E/\pi\omega^2$, where E and ω are pulse energy and beam radius (i.e. $1/e^2$ drop in intensity), respectively. The peak intensities are calculated using the pulse duration of 90 fs and 50 fs for 400 nm and 800 nm.

Supplementary Table 2. Estimated temperature jumps and carrier densities from the effective absorption of the pump pulses. Note that the carrier density is near the electron-hole plasma region for Mott transition (~10^{14} cm^{-2}).[8] Calculated error bars are shown in the parentheses.

	Peak Fluence (mJ per cm^2)	Peak Intensity (GW per cm^2)	Absorption (%)	Absorbed Energy (μJ)	Pump Area (cm^2)	Effective Mass m (gram)	ΔT (K)	Carrier Density ($\times 10^{14}$ cm^{-2})
Monolayer 400 nm	0.6 (0.2)	6.8 (1.8)	4.5 (0.1)	0.15 (0.03)	0.011	4.75×10^{-9}	110 (26)	0.28 (0.07)
	2.2 (0.2)	24.7 (1.8)	3.3 (0.03)	0.39 (0.02)	0.011	4.75×10^{-9}	292 (19)	0.74 (0.05)
	4.2 (0.2)	46.5 (1.8)	2.5 (0.01)	0.55 (0.03)	0.011	4.75×10^{-9}	416 (18)	1.10 (0.05)
Bilayer 400 nm	0.84 (0.2)	9.4 (2.3)	8.0 (0.3)	0.27 (0.06)	0.0082	7.40×10^{-9}	133 (28)	0.68 (0.14)
	1.7 (0.2)	18.8 (2.3)	6.90 (0.16)	0.47 (0.05)	0.0082	7.40×10^{-9}	227 (23)	1.15 (0.12)
	3.4 (0.2)	37.9 (2.3)	5.20 (0.04)	0.72 (0.03)	0.0082	7.40×10^{-9}	350 (19)	1.77 (0.10)
	5.1 (0.2)	56.4 (2.3)	4.20 (0.01)	0.87 (0.04)	0.0082	7.40×10^{-9}	423 (19)	2.14 (0.10)
Bilayer 800 nm	1.0 (0.2)	20.3 (3.1)	2.7 (0.03)	0.18 (0.02)	0.013	11.7×10^{-9}	55 (9)	0.55 (0.09)
	2.0 (0.2)	40.3 (3.1)	2.6 (0.05)	0.34 (0.03)	0.013	11.7×10^{-9}	105 (11)	1.1 (0.10)
	4.0 (0.2)	80.2 (3.1)	2.5 (0.11)	0.64 (0.04)	0.013	11.7×10^{-9}	197 (17)	2.0 (0.17)

Supplementary Note 2. Temperature Jumps from Non-radiative Relaxation to Lattice Disorder.

Temperature jumps of UED measurements are calculated by using mean-square displacements obtained from Debye-Waller responses and equation (3) to (5) in the main context. These results and estimated temperature jumps from effective absorption estimated in Supplementary Note 1 are both shown in Supplementary Table 3. A correlation plot between UED and estimated temperatures is displayed in Figure 5 in the main context.

Supplementary Table 3. Temperature jumps from UED measurements and effective absorption of the pump fluences at 400 nm and 800 nm. Estimated error bars are shown in parentheses.

	Peak Fluence (mJ per cm^2)	Effective Carrier Density (cm^{-2})	Mean-Square Displacement (Å^2)	ΔT_{UED} (K)	ΔT_{abs} (K)
Monolayer 400 nm	0.6 (0.2)	2.8×10^{13}	$4.5\ (0.5)\times10^{-3}$	93 (10)	110 (26)
	2.2 (0.2)	7.4×10^{13}	$15.6\ (0.7)\times10^{-3}$	322 (14)	292 (19)
	4.2 (0.2)	1.1×10^{14}	$20.1\ (0.8)\times10^{-3}$	414 (17)	416 (18)
Bilayer 400 nm	0.84 (0.2)	6.8×10^{13}	$5.6\ (0.2)\times10^{-3}$	116 (4)	133 (28)
	1.7 (0.2)	1.2×10^{14}	$9.8\ (0.3)\times10^{-3}$	203 (7)	227 (23)
	3.4 (0.2)	1.8×10^{14}	$13.0\ (0.4)\times10^{-3}$	269 (9)	350 (19)
	5.1 (0.2)	2.1×10^{14}	$17.4\ (1.2)\times10^{-3}$	359 (25)	423 (19)
Bilayer 800 nm	1.0 (0.2)	$5.5\ \times10^{13}$	$2.0\ (0.2)\times10^{-3}$	42 (4)	55 (9)
	2.0 (0.2)	1.1×10^{14}	$4.0\ (0.2)\times10^{-3}$	82 (4)	105 (11)
	4.0 (0.2)	2.0×10^{14}	$8.3\ (0.3)\times10^{-3}$	172 (6)	197 (17)

Supplementary Note 3. Kinetic Plots of Bilayer $MoSe_2$ at the Lowest and Highest Carrier Densities.

Pump-probe kinetic plots of bilayer $MoSe_2$ at a carrier density of 0.68×10^{14} cm^{-2} are shown in Supplementary Figure. 2(a). Each trace is fitted with single exponential function convoluted

with instrumental function of MeV-UED system. The pump fluence is 0.84 mJ/cm^2. Similar to the results of bilayer at 1.8×10^{14} cm^{-2}, sub-picosecond dynamics are observed. The error bar shown here depicts 68% confidence interval from multiple scan. At highest carrier doping (2.1×10^{14} cm^{-2}), we observed a complex decay in diffraction intensities. Supplementary Figure 2(b) displays the kinetics plots of atomic thin $MoSe_2$ bilayer at carrier density of 2.1×10^{14} cm^{-2} for {100}, {200} {210} and {220} diffraction planes. Single exponential decay function used in the lower carrier densities cannot fit these experimental results with a sub-picosecond time constant. Instead, a biexponential decay function for Bragg peak intensity fitting are employed at this carrier density. The sub-picosecond change of kinetics are supported by our phonon softening model, which shows an ultrafast electron-phonon energy transfer as described in the main context.

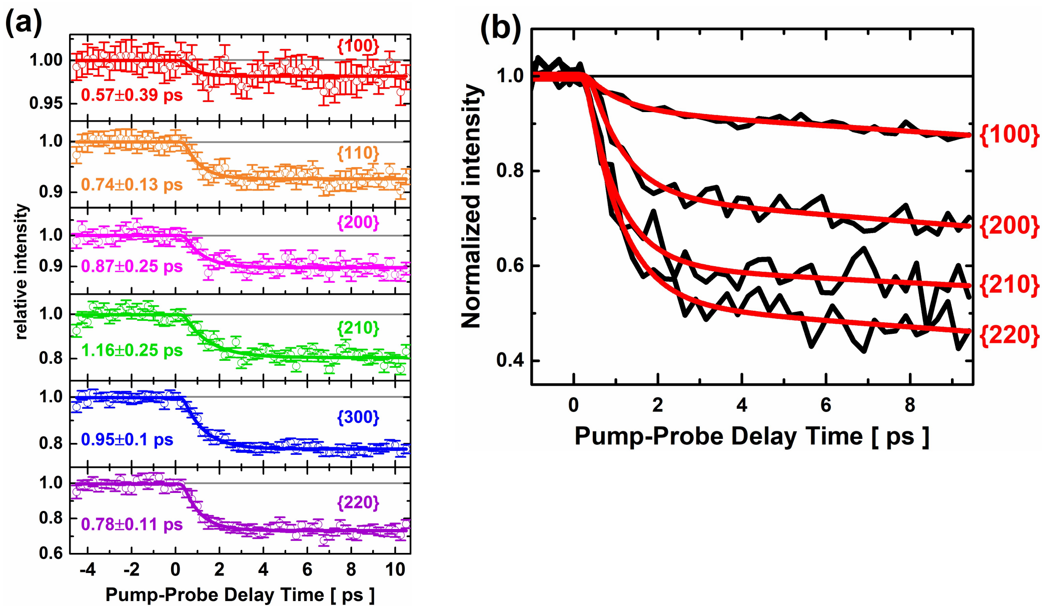

Supplementary Figure 2 | Pump-probe kinetics at the lowest and highest carrier densities.

(a) Kinetic plots at a carrier density of 0.68×10^{14} cm^{-2}. Each trace is fitted to a single exponential function convoluted with UED temporal resolution (~200 fs). The resulted time constants are all in sub-picosecond regime for carrier density below 1.8×10^{14} cm^{-2}. (b) Kinetic plots at a carrier density of 2.1×10^{14} cm^{-2} for

several diffraction planes. Experimental results are fit temporally with a biexponential function convoluted with instrumental response function of UED system.

Supplementary Note 4. Timescale for Lattice Thermalization.

The equilibrated lattice temperature is also supported by our NAQMD simulations. Figure 3(b) in the main text shows the evolution of lattice temperature with simulation time, which indicates that the lattice temperature saturates at a value of $T = 350$ K after an initial evolution period of $t = 0.5$ ps to 1 ps. Supplementary Figure 3 below shows histograms of atomic velocities in the NAQMD simulation cell at times $t = 0$ and 5 ps, respectively, showing that the lattice is in a non-thermal state with multiple velocity peaks at $t = 0$ but evolves to a Maxwell-Boltzmann-like peak distribution at times near ~5 ps.

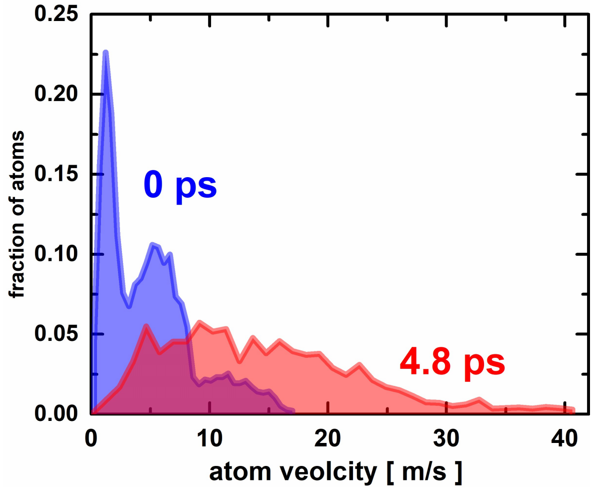

Supplementary Figure 3 | Atomic velocity distributions.

Histograms of atomic velocity (meter per second) in the NAQMD simulation cell at two representative times, t = 0 immediately after electronic excitation, and t = 4.8 ps after reaching a steady state temperature of 350 K. The rapid decay of the initial non-thermal distribution of atomic velocities to a more thermalized distribution supports the assignment of a unique lattice temperature after photoexcitation.

Supplementary Note 5. Power Dependence Measurements and Kinetics Plots for Monolayer $MoSe_2$.

Supplementary Figures 4(a) and 4(b) show pump-probe kinetics of $MoSe_2$ monolayer for {110} and {300} families, respectively, as a function of pump fluences at 400 nm. Ultrafast lattice disorders are observed with time constants in the range from 0.5 ps to 0.8 ps. The time constants do not show any carrier density dependence as observed for bilayer at 400 nm and 800 nm photoexcitations. Large error bars of time constant in {300} family originates from low diffraction intensity at high *Q*. Supplementary Figure 4(c) displays Debye-Wall responses with corresponding linear least squares fits at three effective carrier density ranging from 2.8×10^{13} cm^{-2} to 1.1×10^{14} cm^{-2} (i.e. pump fluence from 0.62 mJ per cm^2 to 4.2 mJ per cm^2).

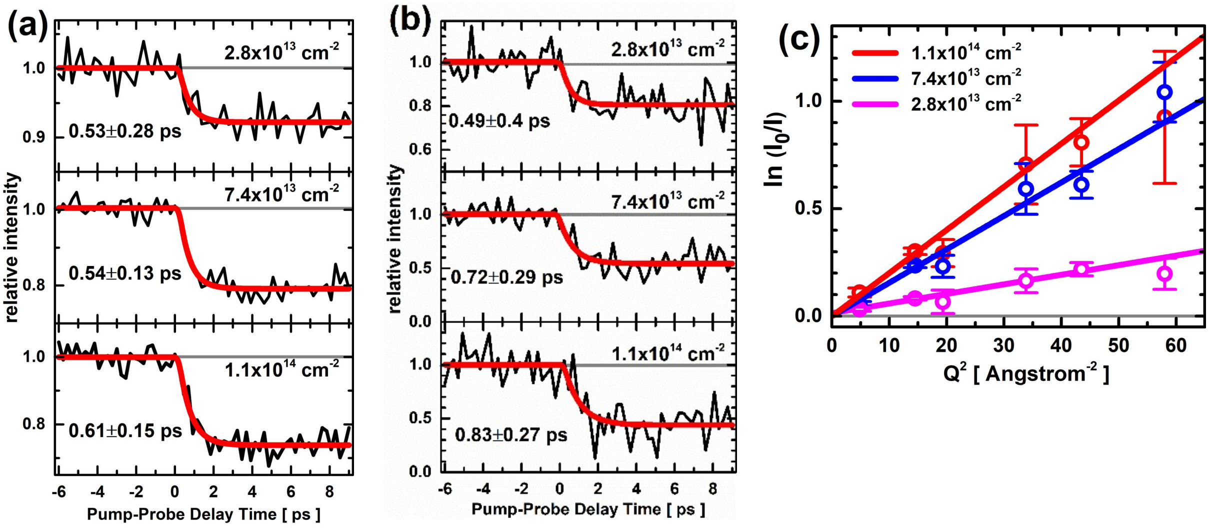

Supplementary Figure 4 | Pump-probe kinetics and Debye-Waller responses for monolayer $MoSe_2$. Time resolved diffraction intensity change of monolayer $MoSe_2$ as a function of pump-probe delay time at three carrier densities. (a) Diffraction peaks from {110} family and (b) diffraction peaks from {300} family with increasing carrier densities toward bottom. The time scale at depletion edge reveals an ultrafast lattice disorder within ~1 ps. (c) Debye-Waller responses with linear least square fits of six diffraction families at

each carrier density. From these slopes, we are able to extract the mean-square displacements and temperature jumps induced by 400 nm for monolayer $MoSe_2$.

Supplementary Note 6. NAQMD simulations on $MoSe_2$ monolayers.

Supplementary Figure 5 summarizes results obtained from NAQMD and DFT simulations on $MoSe_2$ monolayers which shows qualitatively similar Debye-Waller factor (DWF) decays and phonon softening behavior observed in bilayer $MoSe_2$ experiments and simulations described in the main text. Simulations are performed on a 6×6×1 monolayer unit cell of monolayer $MoSe_2$ crystal containing 108 atoms with parameters identical to ones described in the main text. Calculated DWF values for the {110} and {300} planes of the $MoSe_2$ monolayer in Supplementary Figure 5(a) show ultrafast decrease in relative intensity indicating structural disordering on a picosecond timescale. Supplementary Figures 5(c) to 5(f) show the progressive destabilization of the potential energy surface of $MoSe_2$ with increasing charged carrier concentration leading to structural instabilities at the M- and K- points in q-space at an effective charge carrier concentration of 3×10^{14} cm^{-2} whose eigenvectors are depicted in Supplementary Figure 5(b).

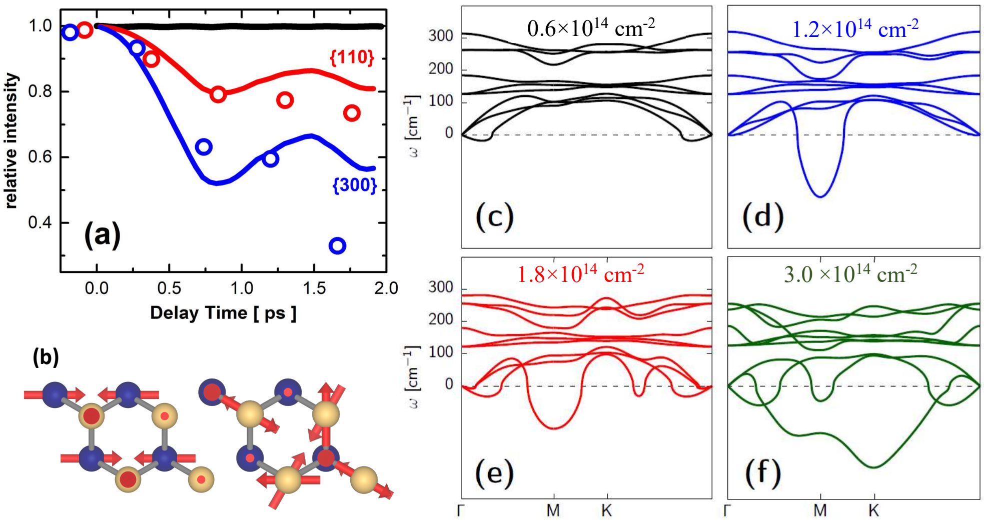

Supplementary Figure 5 | NAQMD simulation results for monolayer $MoSe_2$.
(a) Debye-Waller factor for the $MoSe_2$ monolayer as a function of delay time for the {110} and {300} planes calculated from NAQMD simulations at carrier density of 0 cm^{-2} (black line) and 2.0×10^{14} cm^{-2} (solid lines), and with experimental results (circles), respectively. This demonstrates that photoexcitation results in a marked increase in structural disorder and a reduction in the Bragg peak intensity within a picosecond. (b) Eigenvectors for the soft acoustic modes at the M-point and K-point, respectively. Red arrows (dots) represent in-plane (out-of-plane) atomic motions for Mo and Se atoms as labelled in blue and yellow colors, respectively. Big (Small) red dots indicates that atom moves toward (away from) readers. (c) to (f) display phonon dispersion curves for the $MoSe_2$ monolayer with the carrier density of (c) 0.6×10^{14} cm^{-2}, (d) 1.2×10^{14} cm^{-2}, (e) 1.8×10^{14} cm^{-2}, and (f) 3.0×10^{14} cm^{-2}, respectively. (d, e) show the formation of a strong soft vibration mode at M-point at low electron-hole pair densities, and (f) the emergence of more electron-phonon coupling channels at higher concentration of excited charge carriers.

Supplementary Note 7. Sample Preparation and Characterization.

The $MoSe_2$ samples were grown using chemical vapor deposition technique from MoO_3 and Se powder as the precursors on Si/SiO_2 substrates.[9] Growth carried out at 750 °C for 20 min in the presence of 100 sccm Ar/H_2 gas yielded high coverage of monolayer triangular flakes as shown in Supplementary Figure 6(a). Increasing the growth temperature yielded bilayer $MoSe_2$ flakes (Supplementary Figure 6(b)). These samples were spun coated with PMMA followed by etching of the underlying SiO_2 films in aqueous KOH solution. The PMMA/$MoSe_2$ film was then transferred on top of 10 nm thick Si_3N_4 TEM grid (i.e. SN100-A10Q33B, SIMPore Inc.) and PMMA was dissolved away in acetone.

The characterization and identification of layer thickness was carried out by Raman and photoluminescence (PL) spectroscopy with 532 nm excitation as shown in Supplementary Figures

6(c) and 6(d). For the monolayer sample the out of-plane Raman mode (A_{1g} mode) appeared at 240.7cm^{-1} which shifted to higher wavenumber (241.7cm^{-1}) for bilayer due to interlayer interaction. The in-plane E^1_{2g} mode appears at higher wavenumbers than A_{1g} in $MoSe_2$. In single layer $MoSe_2$, this mode appeared at 287cm^{-1}, while in bilayer $MoSe_2$ it shifted to 285 cm^{-1} due to stiffening with increase in layer number. The Raman spectrum of the bilayer sample shows an additional peak at 352.5 cm^{-1} which corresponds to the inactive B_{2g} mode as reported to be Raman active in few layer 2H-$MoSe_2$ due to the breakdown of translational symmetry.[10,11] We observe strong photoluminescence (PL) from the monolayer sample with peak at 1.55eV, while the PL is quenched in the bilayer sample with peak position shifted to ~1.45 eV indicating the evolution to an indirect bandgap.

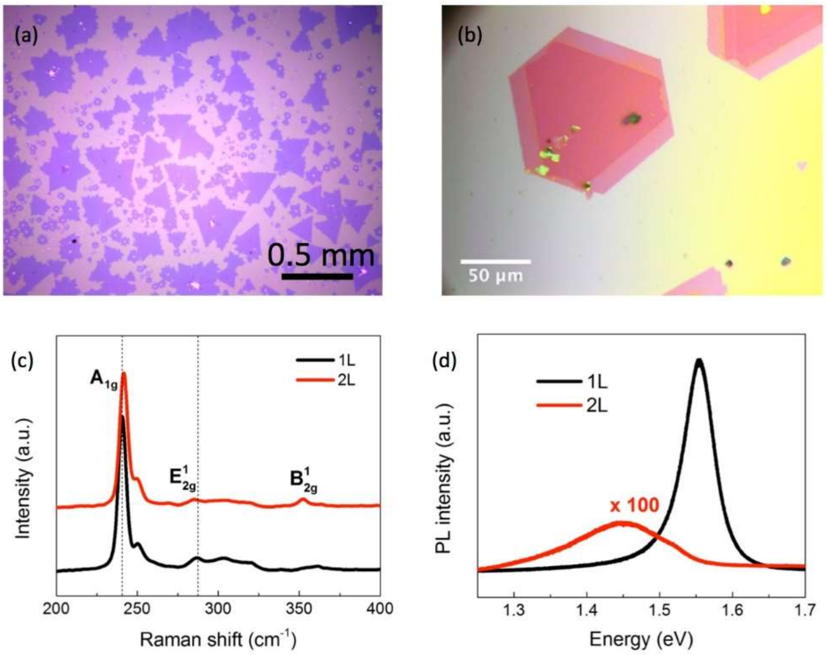

Supplementary Figure 6 | Sample characterizations.

Optical micrographs of CVD grown $MoSe_2$. (a) Single layer and (b) bilayer. (c) Raman spectrum of monolayer (1L) and bilayer (2L) $MoSe_2$, and (d) photoluminescence spectra of 1L and 2L $MoSe_2$ samples.

Supplementary References

1. Heavens, O. S. in *Optical Properties of Thin Solid Films* (Dover Publications, INC, 1965).

2. Liu, H. L. *et al.* Optical properties of monolayer transition metal dichalcogenides probed by spectroscopic ellipsometry. *Appl. Phys. Lett.* **105,** 201905 (2014).

3. Philipp, H. R. Optical Properties of Silicon Nitride. *J. Electrochem. Soc. Solid-State Sci. Technol.* **120,** 295–300 (1973).

4. Beadie, G., Brindza, M., Flynn, R. A., Rosenberg, A. & Shirk, J. S. Refractive index measurements of poly (methyl methacrylate) (PMMA) from 0.4–1.6 μm. *Appl. Opt.* **54,** 139–143 (2015).

5. Morozov, Y. V. & Kuno, M. Optical constants and dynamic conductivities of single layer MoS_2, $MoSe_2$, and WSe_2. *Appl. Phys. Lett.* **107,** 83103 (2015).

6. Wang, K. *et al.* Broadband Ultrafast Nonlinear Absorption and Nonlinear Refraction of Layered Molybdenum Dichalcogenide Semiconductors. *Nanoscale* **6,** 10530–10535 (2014).

7. Kiwia, H. L. & Westrum, E. F. Low-temperature heat capacities of molybdenum diselenide and ditelluride. *J. Chem. Thermodyn.* **7,** 683–691 (1975).

8. Chernikov, A., Ruppert, C., Hill, H. M., Rigosi, A. F. & Heinz, T. F. Population inversion and giant bandgap renormalization in atomically thin WS_2 layers. *Nat. Photonics* **9,** 466–470 (2015).

9. Wang, X. *et al.* Chemical vapor deposition growth of crystalline monolayer $MoSe_2$. *ACS Nano* **8,** 5125–5131 (2014).

10. Tonndorf, P., Schmidt, R., Bottger, P., Zhang, X. & Borner, J. Photoluminescence emission and Raman response of monolayer MoS_2, $MoSe_2$, and WSe_2. *Opt. Express* **21,** 4908 (2013).

11. Lu, X. *et al.* Large-Area Synthesis of Monolayer and Few-Layer $MoSe_2$ Films on SiO_2 Substrates. *Nano Lett.* **14,** 2419–2425 (2014).
